# Aligning front-of-pack labelling with dietary guidelines: including whole grains in the health star rating

**DOI:** 10.1007/s00394-024-03404-z

**Published:** 2024-04-23

**Authors:** Caitlin Byron, Katrina R Kissock, Eden M Barrett, Eleanor J Beck

**Affiliations:** 1 School of Medical, Indigenous and Health Sciences, University of Wollongong, Wollongong, NSW 2522 Australia; 2 The George Institute for Global Health, Sydney, NSW 2000 Australia; 3 School of Health Sciences, University of New South Wales, Sydney, 2052 Australia

**Keywords:** Whole grain, Front of pack labelling, Dietary guidelines, Nutrient profiling, Diet quality

## Abstract

**Purpose:**

Front-of-pack labelling systems, such as the Health Star Rating (HSR), aim to aid healthy consumer dietary choices and complement national dietary guidelines. Dietary guidelines aim to be holistic by extending beyond the individual nutrients of food, including other food components that indicate diet quality, including whole grains. We aimed to test the feasibility of including whole grains in the HSR algorithm, to better inform dietary guidance in Australia coherent with existing dietary guidelines.

**Methods:**

We assigned whole-grain points as a favourable component of the HSR based on the whole-grain content of foods. We compared the original, and three modified HSR algorithms (including altered thresholds for star ratings) using independent-samples median tests. Finally, we used Spearman’s correlation to measure the strength of association between an item’s nutritional composition (all components of the HSR algorithm including all favourable and unfavourable components) and their HSR using each algorithm.

**Results:**

Up to 10 points were added for products with ≥ 50% whole-grain content, with no points for products with < 25%. Adjusting the HSR score cut-off by 3 points for grain products created the greatest difference in median HSR between refined and whole-grain items (up to 2 stars difference), compared to the original algorithm (a maximum of 1 star).

**Conclusions:**

The addition of whole grains to the HSR algorithm improved the differentiation of refined and whole-grain items, and therefore better aligned with dietary guidelines. Holistic approaches to food guidance systems are required to provide consistent messaging and inform positive food choices.

## Introduction

Low whole-grain intake is one of the leading dietary risk factors for death and disability in Australia and globally, accounting for approximately 82 million disability adjusted life years and 3 million deaths globally in 2017 [1]. Whole-grain consumption is associated with a reduction in risk factors for non-communicable diseases, such as abnormal blood lipids and high blood pressure [2–5]. Whole grains include the bran, germ and endosperm of the grain and if processed in any way must include these constituents in a ratio similar to the intact grains [6]. Refining grains results in the loss of the bran and germ, dense in cereal fibre, B vitamins and minerals [7, 8], and hence reduces the beneficial components of the grain. Despite the evidence, Australians consume less than half the recommended daily intake of whole grains (48 g per day) and intake is even lower in some other countries [9].

Front-of-pack labelling (FOPL) systems are tools designed to be easily understood that promote healthier choices in line with dietary guidelines [10]. These systems, sometimes called nutrient profiling, and including the Australian Health Star Rating (HSR), often do not to account for the whole-grain content of foods in their underlying algorithm [11]. However, the benefits of whole grains extend beyond the fibre they provide, and contain additional components such as magnesium and polyphenols [7]. Specifically, review of population dietary intake data identifies cardiovascular benefit of whole-grain intake in addition to benefits noted only for cereal fibre [12, 13]. Therefore, through failure to account for the whole-grain content of food, these systems may fail to communicate the benefits of key whole grains over some higher fibre grains, including some refined grains. This limits the opportunity to promote the whole grains listed in dietary guidelines [11]. Guidelines encourage the consumption of mostly whole grains (Australian Dietary Guidelines (ADG)) or to make at least half of your grains whole grain (Dietary Guidelines for Americans). The HSR algorithm includes positive and negative scoring related to positive attributes of foods protein, fibre, fruits, vegetables, nuts and legumes and negative attributes of energy, sodium, saturated fat and sugars. Although previous consultations have been undertaken to include whole grain in the algorithm, it was argued that the HSR already accounts for whole grain through the attribution of positive points for the fibre content of the food [14]. Yet, other organisations have recognised the contribution of whole grains and they have been successfully incorporated into nutrient profiling in Nordic countries, namely the Keyhole label [15]. The guidelines for the Keyhole labelling are matched to the Nordic Nutrition Recommendations [16].

We have previously modelled inclusion of whole grains into the algorithm underlying the French Nutri-Score FOPL system [17]. This work utilised the suggested minimum whole-grain levels outlined in the Whole Grain Initiative’s (WGI) whole-grain food definition, as cut points in applying positive points for whole-grain content. Applying the original and modified algorithms to food composition databases (including from Australia), we found greater correlations between diet quality scores and the algorithm with the addition of whole grains [17]. The current study aims to model the inclusion of whole grain into the algorithm underlying the HSR (from hereon, HSR algorithm) to demonstrate the feasibility of such a modification and improve its alignment with the recommendations of the ADG for grains and cereal foods. This modification may better differentiate between refined and whole-grain products, encouraging consumers to increase their whole-grain intake.

## Methods

This study encompassed the development and comparisons of modified HSR algorithms that account for the whole-grain content of a food. Both the original and modified systems were applied to nutrient composition databases for Australia. Data on the nutrient composition of food and beverages was extracted from the Australian Nutrient Composition (AUSNUT) 2011-13 database [18]. The Expanded Australian Whole-grain Database, containing data on the whole-grain content (g/100 g) based on the dry weight of items from the AUSNUT 2011-13 database was also used [19].

### Modified HSR

We applied the original HSR algorithm to items in the AUSNUT 2011-13 database using Microsoft Excel (Microsoft 365 MSO, Version 16.0.13426.20306) informed by the HSR guide [20]. In brief, to calculate a star rating, foods/beverages were categorised into one of six categories: (1) Non-dairy beverages, 1D. Milk and Dairy beverages (and alternatives), (2) Foods, 2D. Dairy foods (and alternatives), (3) Oils and Spreads, 3D. Cheese. Category 2 foods are the only foods which contain whole grain and therefore only these foods were modified in this modelling study. HSR ‘baseline points’ were calculated using components linked with increased risk of non-communicable diseases. This includes energy (kJ) and total sugars (g) content (for Category 1), as well as saturated fat (g) and sodium (mg) content (for the remaining categories), per 100 g/ml of the food item.

‘Modifying points’ were then allocated in relation to the percentage of fruits, vegetables, nuts and legumes (FVNL) of a product, referred to as ‘HSR V points’. These points are calculated based on the concentrated and/or non-concentrated FVNL content of a food. There are no clear criteria of what ingredients can contribute to the concentrated or non-concentrated FVNL of a food. To ensure consistency, we used the HSR guide [20] and the “Example of potential additional guidance on eligibility for FVNL and concentrated FVNL” from the HSR Technical Advisory Group [21], when calculating the “V points” of food items. The Australian Healthy Survey – Australian Dietary Guidelines (AHS-ADG) database [22] and the AUSNUT 2011-13 food recipe file [23] were used to determine the concentrated and non-concentrated FVNL content of each food and the equation provided in the HSR guide [20] was used to calculate the FVNL percentage foods containing both concentrated and non-concentrated FVNL. The AHS-ADG is based on estimations, therefore, some items received greater than 100% FVNL content. These products were assigned the maximum V points.

Categories 1D-3D may also receive additional modifying points for protein, ‘HSR P points’ or dietary fibre, ‘HSR F points’ content, which were calculated. Point allocation for each food is dependent on its categorisation. Additional considerations outlined in the HSR guide were also applied, for example, not assigning ‘P points’ to items with greater than 12 baseline points, unless they obtained five of more ‘V points’. These modifying points were then subtracted from the baseline points to produce a HSR score, which is coherent to a HSR rating [20].

Cut-offs are then applied to the total score to determine the number of stars a product is awarded. Here, the original HSR algorithm was modified by including and assigning modifying points for the whole-grain content of food items, ‘WG points’. According to the WGI, foods with ≥ 25% whole-grain ingredients are eligible for stating the presence of whole grains on FOPL, and a food can only be labelled as a ‘whole-grain food’ if it is composed of a ≥ 50% whole-grain ingredients, based on dry weight [24]. These percentage values were used to determine the WG points an item could score based on their whole-grain content. The method of allocating WG points was modelled on previously trialled methods [17]. Foods containing 25–100% whole grain scored up to 10 WG points, with higher percentages equating to more points (Table 1). The 25% whole-grain cut-off approximately correlates to 8 g whole grain per serve, which was the minimum amount used in early studies to show the relationship between whole-grain intake and positive health outcomes [25]. No points were applied for foods below 25%, as this is widely considered the minimal acceptable amount to make an impact on whole-grain intake and the higher cut-off may encourage manufacturers to produce items with a greater whole-grain content [24].

**Table 1:** Whole-grain cut-off points to create modified HSR algorithm

Points	0	1	2	3	4	5	6	7	8	9	10
Whole-grain percentage (dry weight)^**a**^	< 25		≥ 25				≥ 50				100

Higher points are indicative of better nutritional quality

^a^Whole-grain percentage cut-offs are derived from the Whole Grain Initiative recommendations

The whole-grain content of items was determined, using the whole-grain values from the Expanded Australian Whole-grain Database. WG points were applied to the HSR score equation in the same way as the other modifying points. Category 2 foods in the HSR system includes all foods other than beverages, dairy and oils, therefore the whole-grain points were applied only to grain foods in Category 2, which in effect, was only grain foods which contained ≥ 25% whole-grain ingredients. The following formula was used to determine HSR score.

Equation 1

*HSR score = HSR baseline points – (V points*^*a*^*+ P points*^*b*^*+ F points*^*c*^*+ WG points*^*d*^*)*.

Equation 1: Modified HSR equation to determine the HSR score of an item.

^a^Points for fruits, vegetables, nuts and legumes content.

^b^Points for protein content.

^c^Points for fibre content.

^d^Points for whole-grain content.

Initial models indicated that modifying only the points assigned to foods, but maintaining the same cut-offs for scores to award the stars tended to poorly differentiate between items with differing amounts of whole-grain content and nutrition composition. Therefore, to account for the addition of ‘WG points’, different ranges to assign HSR scores to a rating were trialled (Table 2). We compared shifting cut off ranges by 3 points or 10 points (maximum as we had added up to 10 whole grain points) to consider methods to maximise the differentiation between refined and whole-grain foods. Similar to the application of modified points, in this comparison food items were considered ‘whole-grain’ items if they contained ≥ 25% whole-grain ingredients and the rest as ‘refined grain’ items.

**Table 2:** Health Star Rating (HSR) score cut-offs and corresponding final HSR for Category 2 gra in foods

HSR	original^a^	shift by -3^b^	shift by -10^c^
5.0	≤-11	≤-14	≤-21
4.5	-10–7	-13–10	-20–17
4.0	-6–2	-9–5	-16–12
3.5	-1–2	-4–1	-11–8
3.0	3–6	0–3	-7–4
2.5	7–11	4–8	-3–1
2.0	12–15	9–12	2–5
1.5	16–20	13–17	6–10
1.0	21–24	18–21	11–14
0.5	≥ 25	≥ 22	≥ 15

^a^Score cut-offs are the same as those used in the original algorithm

^b^Score cut-offs are 3 less than those used in the original algorithm

^c^Score cut-offs are 10 less than those used in the original algorithm

To ensure non-grain containing items were not unnecessarily shifted down in star ratings the algorithm was only applied to major grain containing groups, including “cereals and cereal products” (Group 12 in the AUSNUT database), “cereal based products and dishes” (Group 13) and “confectionary and cereal/nut/fruit/seed bars” (Group 28). Group 28 consists of items with and without grains (whole and refined). Non-grain containing items were identified and excluded using the AUSNUT 2011-13 food recipe file [23] and professional judgement. For example, if flour was in an item’s ingredient list on the recipe file, it was considered a grain containing item and was included. Each cut-off test was applied to each grain containing food and analysed within major food groups (those in the database represented on a 2-digit level – e.g. cereals and cereals products is Group 12), as specified in the AUSNUT database. Frequency tables were created on Microsoft Excel (Microsoft 365 MSO, Version 16) to compare the shift in star rating within each major group when applying each modification.

To further examine the shift in HSR, between whole-grain and refined grain foods, we also considered foods at a sub-major group (3 -digit) level. “Regular breads, and bread rolls” (Group 122), “English-style muffins, flat breads, and savoury and sweet breads” (Group 123), “Breakfast cereals, ready to eat” (Group 125) and “Breakfast cereals, hot porridge style” (Group 126), contain items that contribute the most to whole-grain intake in Australia [9], therefore, these groups were combined based on similarities (i.e. 122 and 123, 125 and 126) and analysed in the same way as the 2-digit groups. Hereafter the grouping “Regular breads, and bread rolls” (Group 122) and “English-style muffins, flat breads, and savoury and sweet breads” (Group 123) is referred to as “Bread items” and the grouping of “Breakfast cereals, ready to eat” (Group 125) and “Breakfast cereals, hot porridge style” (Group 126) as “Breakfast cereals”.

### Statistical analysis

Statistical analysis was conducted on IBM SPSS Statistics Version 28. Data was checked for normality using Kolmogorov-Smirnov and descriptive statistics were used to determine the median, interquartile range (IQR) (for non-normally distributed data) and range of whole-grain content (per 100 g) and HSR for whole-grain and refined grain items for each major and sub-major group specified above. Independent-samples median tests were conducted to compare differences in HSR between refined and whole-grain items in each group. Two commonly consumed items (one refined and one whole-grain) from each major group were selected as examples to compare and convey the changes in HSR when applying the different algorithms. This included a refined and whole-grain version of a bread roll (Group 12), a savoury biscuit (Group 13) and a muesli bar (Group 28).

Spearman’s correlation was then used to measure the strength of the association between components of an item’s nutrient composition (whole grain, fibre, protein, fruit/vegetable/nuts/legumes, energy, saturated fat, total sugar and sodium) and the item’s HSR using each algorithm. A *p*-value of < 0.05 was used to determine statistical significance of the component contributing to overall HSR.

## Results

A total of 1557 items made up the three major groups examined, including 499 “cereals and cereal products”, 913 “cereal-based products and dishes” and 143 “confectionery and cereal/nut/fruit/seed bars”. Following exclusion of non-grain containing “confectionery and cereal/nut/fruit/seed bars” (*n* = 100), a total of 1455 items remained. According to the Kolmogorov-Smirnov tests, data in all groups were not normally distributed.

The difference in median HSR between refined and whole-grain items using the original algorithm was 0.5-1.0 star across all major and sub-major groups examined (Table 3). Differences in median HSR between refined and whole-grain items tended be greatest when using the “WG – original” and “WG – shift by -3” algorithms, with differences of 1.0-1.5 stars and 1.0–2.0 stars, respectively. When using the “WG – shift by -3” algorithm, the median HSR for refined grain items in all groups was 0.5 stars lower than the median HSR when using the “original” algorithm. Further, the median HSR for whole-grain items when using the “WG – shift by -3” was greater or equal of that when using the “original” algorithm in all groups except “confectionery and cereal/nut/fruit/seed bars”. For discretionary items of “confectionery and cereal/nut/fruit/seed bars”, the median HSR of whole-grain items was greater when using the “WG – original” algorithm, compared to when using the “original algorithm”. The chosen model of WG – shift by -3, creates the greatest differentiation of HSR for core foods such as breads and breakfast cereals without allowing category shifts upward (more stars) for discretionary items.

**Table 3:** Difference in median HSR between whole-grain and refined grain products using different HSR algorithms

Group	Algorithm	Whole grain			Refined Grain			Difference in medians (*p*^a, b^)
		Median (HSR)	IQR	Range	Median (HSR)	IQR	Range	
***12 – Cereals and cereal products***	Original	4.0	4.0-4.5	1.5-5.0	3.5	3.0–4.0	2.0–5.0	0.5 (< 0.001*)
	WG - original	4.5	4.5-5.0	2.0–5.0	3.5	3.0–4.0	2.0–5.0	1.0 (< 0.001*)
	WG - shift by -3	4.5	4.0–5.0	1.5-5.0	3.0	2.5–3.5	1.5-5.0	1.5 (< 0.001*)
	WG - shift by -10	3.5	3.0–4.0	1.5-5.0	2.5	2.0-2.5	0.5–4.5	1.0 (< 0.001*)
*122&123 – Bread items*	Original	4.0	4.0-4.5	2.0-4.5	3.5	3.0-3.5	1.5-4.0	0.5 (< 0.001*)
	WG - original	4.5	4.0–5.0	2.0–5.0	3.5	3.0-3.5	1.5-4.0	1.0 (< 0.001*)
	WG - shift by -3	4.0	4.0-4.5	2.0–5.0	3.0	2.5–3.5	1.5-4.0	1.0 (< 0.001*)
	WG - shift by -10	3.5	3.0–4.0	1-4.5.0	2.5	2.0-2.5	0.5-3.0	1.0 (< 0.001*)
*125&126 – Breakfast cereals*	Original	4.0	3.5–4.5	1.5-5.0	3.0	2.0–4.0	1.5-5.0	1.0 (0.103)
	WG - original	4.5	4.5-5.0	2.0–5.0	3.0	2.0–4.0	1.5-5.0	1.5 (0.0001*)
	WG - shift by -3	4.5	4.0–5.0	1.5-5.0	2.5	2.0–4.0	1.5–4.5	2.0 (< 0.0001*)
	WG - shift by -10	3.5	3.0–4.0	1.0–5.0	2.0	1.0–3.0	0.5-4.0	1.5 (0.0004*)
***13 - Cereal based products and dishes***	Original	3.5	2.5-4.0	1.0-4.5	2.5	1.5–3.5	0.5–4.5	1.0 (0.017)
	WG - original	4.0	3.0-4.5	2.0–5.0	2.5	1.5–3.5	0.5–4.5	1.5 (0.006)
	WG - shift by -3	3.5	2.5–4.5	1.5-5.0	2.0	1.5-3.0	0.5–4.5	1.5 (0.002*)
	WG - shift by -10	2.5	3.0-3.5	0.5-4.0	1.5	0.5–2.5	0.5–3.5	1.0 (0.004)
***28 – Confectionary & cereal/nut/ fruit/seed bars***	Original	3.0	2.5–3.5	2.5–4.5	2.0	1.5–2.5	0.5-4.0	1.0 (0.014)
	WG - original	3.25	3.0–4.0	2.5-5.0	2.0	1.5–2.5	0.5-4.0	1.25 (< 0.001*)
	WG - shift by -3	2.75	2.5-4.0	2.0-4.5	1.5	1.0–2.0	0.5–3.5	1.25 (< 0.001*)
	WG - shift by -10	2.25	1.5-3.0	1.5–3.5	1.0	0.5–1.5	0.5-3.0	1.25 (0.002*)

^a^2-digit groups: significant *p* ≤ 0.002; ^b^3-digit groups: significant *p* ≤ 0.0004

*Statistically significant

Table 4 presents the shifts in HSR when applying the original compared to the modified algorithms for examples from each major food group. In each group, the “WG – original” and “WG – shift by -3” algorithms both increase the HSR of the whole-grain items compared to the original algorithm by 0.5-1.0 stars, while the “WG – shift by -3” algorithm decreases the HSR of refined items by 0.5 stars. Results for the “WG – shift by -10” algorithms did not provide improved differentiation.

**Table 4:** Shifts in HSR in example food items when applying the different HSR algorithms

Item	Original	WG -original	WG -shift by -3	WG -shift by -10
***12 – Cereals and cereal products***				
**Whole grain**: Bread roll, from wholemeal flour, commercial, toasted	4.0	5.0	5.0	4.0
**Refined grain**: Bread roll, from white flour, commercial, toasted	3.5	3.5	3.0	2.5
***13 - Cereal based products and dishes***				
**Whole grain**: Biscuit, savoury, from wholemeal wheat flour, crispbread	4.0	5.0	4.5	3.5
**Refined grain**: Biscuit, savoury, from wheat flour, crispbread, puffed & toasted	3.5	3.5	3.0	2.0
***28 – Confectionary and cereal/nut/fruit/seed bars***				
**Whole grain**: Bar, muesli or snack, with 20% dried fruit & 5% nuts	4.0	4.5	4.5	3.5
**Refined grain** Bar, muesli or snack, with 15% dried fruit & 25% nuts, added vitamins B1, B2, B3, C & folate, Fe, & Zn	2.5	2.5	2.0	1.5

### Correlations between components and HSR

In general, and as expected, positive correlations were observed between favourable components (whole grain, fibre, protein and FVNL) and overall HSR, and negative correlations between unfavourable components (energy, saturated fat, total sugar and sodium) and overall HSR across groups (Table 5). Interestingly, energy showed positive correlations in the “cereals and cereal products” group, and FVNL showed negative correlations in the “bread items” group, however these were non-significant (*p* > 0.05). While the addition of whole grain to the HSR algorithm strengthened correlations with whole grain, most prominently in the “cereals and cereal products” group, there was little to no change in correlation among other components. The change in correlation for whole grain was similar across the different modifications to the HSR algorithm. This demonstrates the relevance of whole grain as a separate component to fibre in the HSR algorithm.

**Table 5:** Correlation between health star rating (HSR) of grain-based food items with individual components of the HSR algorithm

Group	Algorithm	Correlation between nutritional component and HSR^a, b^							
		Whole grain	Fibre	Protein	FVNL	Energy	Saturated fat	Total sugar	Sodium
***12 – Cereals and cereal products***	Original	0.52*	0.64*	0.45*	0.08	0.03	-0.10*	-0.14*	-0.38*
	WG - original	0.75*	0.64*	0.39*	0.11*	0.02	-0.07	-0.05	-0.37*
	WG - shift by -3	0.73*	0.66*	0.41*	0.12*	0.05	-0.07	-0.05	-0.38*
	WG - shift by -10	0.74*	0.64*	0.42*	0.09*	0.04	-0.09*	-0.06	-0.37*
*122&123 – Bread items*	Original	0.63*	0.79*	0.56*	0.01	-0.22*	-0.31*	-0.39*	-0.27*
	WG - original	0.77*	0.82*	0.55*	-0.07	-0.25*	-0.32*	-0.35*	-0.19*
	WG - shift by -3	0.74*	0.81*	0.53*	-0.04	-0.24*	-0.34*	-0.34*	-0.22*
	WG - shift by -10	0.75*	0.81*	0.55*	-0.05	-0.23*	-0.33*	-0.34*	-0.20*
*125&126 – Breakfast cereals*	Original	0.46*	0.77*	0.51*	0.17*	-0.28*	0.16	-0.43*	-0.40
	WG - original	0.66*	0.69*	0.43*	0.24*	-0.31*	0.23*	-0.41*	-0.44*
	WG - shift by -3	0.69*	0.68*	0.44*	0.25*	-0.28*	0.23*	-0.46*	-0.46*
	WG - shift by -10	0.67*	0.69*	0.44*	0.18*	-0.32*	0.16*	-0.49*	-0.42*
***13 - Cereal based products and dishes***	Original	0.34*	0.08*	0.32*	0.39*	-0.84*	-0.82*	-0.71*	-0.11*
	WG - original	0.36*	0.09*	0.32*	0.38*	-0.84*	-0.82*	-0.71*	-0.11*
	WG - shift by -3	0.36*	0.08*	0.32*	0.38*	-0.84*	-0.81*	-0.70*	-0.12*
	WG - shift by -10	0.36*	0.09*	0.32*	0.37*	-0.81*	-0.79*	-0.70*	-0.10*
***28 – Confectionary & cereal/nut/fruit/seed bars***	Original	0.61*	0.85*	0.18	0.27	-0.41*	-0.78*	-0.65*	-0.38*
	WG - original	0.66*	0.87*	0.16	0.25	-0.41*	-0.77*	-0.66*	-0.34*
	WG - shift by -3	0.66*	0.88*	0.21	0.25	-0.36*	-0.71*	-0.67*	-0.39*
	WG - shift by -10	0.66*	0.89*	0.17	0.27	-0.35	-0.71*	-0.67*	-0.37*

*Statistically significant (*P* < 0.05)

a. Spearman’s correlation coefficients

b. Component content units: whole grain (g dry weight); fibre (g); protein (g); Fruit/vegetable/nuts/legumes (FVNL) (%), energy (kJ), saturated fat (g), total sugar (g), sodium (mg)

## Discussion

FOPL systems and nutrient profiling, such as the HSR, are intended to complement dietary guidelines by promoting healthier choices, including the choice of whole grain over refined grain. However, it is evident that the current HSR system fails to effectively distinguish between refined and whole-grain items and hence communicate the benefits of consuming mainly whole grains. Our findings demonstrate that the addition of whole-grain content to the HSR algorithm better differentiates between refined and whole-grain items. Across all groups, the smallest difference in median HSR between refined and whole-grain items was observed when using the original algorithm. On the other hand, the use of the “WG – shift by -3” algorithm led to the greatest difference between refined and whole-grain items across all groups. This was less evident in the grain containing snack items of confectionary and cereal/nut/fruit/seed bars, however considering these are discretionary foods in the Australian Dietary Guidelines this could be considered a positive of this model. That is “WG – shift by -3” was chosen as the preferable modification as it does not create further anomalies between systems and recommendations by encouraging ‘discretionary’ items.

The modified algorithm, resulting in an HSR that more clearly promoted whole grains over refined grains, would better align with the ADG and of course the body of evidence showing increased whole grain intake is associated with a multitude of health benefits. Such changes in algorithms potentially aid in guiding individuals to increase whole-grain intake in Australia. Positive changes to whole-grain intake were seen with the introduction of whole grains into the Scandinavian Keyhole label system in 2009. The Danish Whole Grain Partnership aims to increase population whole-grain intake and has contributed to a 76% whole-grain intake increase in Denmark [26]. One strategy of this partnership is the labelling of products high in whole grain using the minimum whole-grain requirements set by the Keyhole system. Therefore, to display the partnership’s whole-grain logo (a bright orange logo linked with the choose whole grains first slogan) or the Keyhole label manufacturers are encouraged to produce items in a way that meets minimum whole-grain requirements. For example, breakfast cereals must contain ≥ 65% whole-grain ingredients [26]. Although Danish whole-grain intake improvements were not solely due to the introduction of whole grains to the Keyhole system, it is likely to have made a considerable contribution. For example, there are a growing number of Keyhole labelled products on the market, with over 4000 items in Danish supermarkets and 94% of Danes understanding the benefits of choosing these items over non-labelled items [27]. A greater proportion of the population also meet or exceed recommendations for whole-grain intake [28].

Although the changes in HSR may seem small, they may make considerable impacts on consumer choices, particularly at a food item level. For example, when comparing a wholemeal bread roll to a white flour bread roll, the initial difference in HSR was 0.5 stars (4 vs. 3.5 stars). Modifying the algorithm to include whole grains and then shifting the star cut-offs by 3, as seen in “WG – shift by -3”, led to a greater differentiation of 2 stars (5 vs. 3 stars). This is particularly relevant for consumers, as there is a lack of knowledge towards, and a high level of consumer scepticism of the health benefits of choosing whole grains [29]. Studies show that consumers face uncertainty when interpreting ‘mid-range’ HSR, often unable to decide if the product is ‘healthy’ or not [30]. Our findings, along with others, reveal that many grain products are awarded between 3 and 5 stars [31]. Therefore, the greater differences in HSR between refined and whole-grain items, as presented above, are more likely to have a substantial effect in promoting and encouraging consumers to choose whole-grain over refined options. Critically, front-of-pack labelling to encourage improved dietary choices may be most effective if industry is willing to adopt these systems. At present the HSR is not mandatory and slightly skewed towards foods that rate highly, with researchers evaluating HSR highlighting a need to make the system mandatory to achieve maximum effect [32].

Possible contentions against our modified HSR algorithm are that it does not change the scoring of other nutrient components (energy, sodium, fibre, etc.). Consequently, the addition of whole grains to the algorithm may weaken the effect that other components have on the final HSR. However, our results show that the use of the modified algorithm resulted in little to no changes to the correlations between original HSR components and overall HSR. A similar finding was observed in the study by Kissock et al. where the addition of whole grain to the Nutri-score front of pack labelling algorithm only slightly altered correlations between Nutri-score rankings and other nutrient components [17].

Additionally, some may argue that the modified algorithm will double count cereal fibre from the fibre points and whole-grain points. However, the benefits of whole grains go beyond the fibre they provide. A study exploring the associations between risk factors of CVD with whole grain and cereal fibre intake found that whole-grain intake was still associated with reduced CVD risk factors after adjustment for cereal fibre [7, 12, 13]. Furthermore, the whole-grain content of a food does not always align with its fibre content. Some refined grain products contain a greater fibre content than whole-grain products or contain added fibre. As a result, it is likely that the current HSR of refined grain items would be similar or greater than that of whole-grain items. For example, “Bread, from white flour, commercial, added fibre” and “Bread, from wholemeal flour, commercial” both have a fibre content of 6.1 g per 100 g. Despite the whole-grain item having a far greater whole-grain content than the refined grain, both items receive a HSR of 4. When applying the modified algorithm, they differ by 1 star. Therefore, adding whole grains to the HSR algorithm, rather than using fibre as a surrogate for whole grains, better promotes foods containing substantial whole grain. It also better adheres to national dietary guidelines, which promote both fibre and whole-grain intake [33].

A limitation of this study may be around the use of the AUSNUT database foods which is now a decade old. However, this work is a comparison, and therefore the principle point of how HSR can better highlight whole-grain foods is demonstrated, particularly given the large number of foods/whole-grain foods considered. The development of the Expanded Australian Whole-grain Database was met by several limitations, including using assumptions to estimate values and trying to account for variations in the whole-grain content of some items due to factors such as the level of processing an item undergoes [34]. Similar limitations may be considered for calculation the HSR and estimating fruit, vegetable, nut and legume content. Furthermore, our study did not explore the effect of modifying other components of the HSR algorithm concurrently, and potentially review of a single component of a food requires extensive review of all positive and negative attributes of a food. However, considering the whole-grain modification against the other components of the algorithm somewhat alleviates this issue.

Our findings demonstrate the feasibility of incorporating whole grains into the HSR algorithm, where these modifications may better align with the ADGs, and clearly promote and differentiate whole-grain over refined-grain products. It is thought that consistent messaging between the ADG and HSR will reduce consumer confusion, particularly with grain-based products, and encourage the development or refinement of future food guidance systems to be coherent with existing systems and guidelines.

## Data Availability

Data described in the manuscript will be made available (where possible) upon request. Raw data relating to the Australian National Nutrition and Physical Activity Survey 2011-12 requires access approval by the Australian Bureau of Statistics.

## Code availability

Code used in the statistical analysis will be made available (where possible) upon request.
